# Preclinical models for obesity research

**DOI:** 10.1242/dmm.026443

**Published:** 2016-11-01

**Authors:** Perry Barrett, Julian G. Mercer, Peter J. Morgan

**Affiliations:** The Rowett Institute of Nutrition and Health, University of Aberdeen, Foresterhill, Aberdeen AB25 2ZD, UK

**Keywords:** Binge eating, Epigenetics, Obesity, Transgenics

## Abstract

A multi-dimensional strategy to tackle the global obesity epidemic requires an in-depth understanding of the mechanisms that underlie this complex condition. Much of the current mechanistic knowledge has arisen from preclinical research performed mostly, but not exclusively, in laboratory mouse and rat strains. These experimental models mimic certain aspects of the human condition and its root causes, particularly the over-consumption of calories and unbalanced diets. As with human obesity, obesity in rodents is the result of complex gene–environment interactions. Here, we review the traditional monogenic models of obesity, their contemporary optogenetic and chemogenetic successors, and the use of dietary manipulations and meal-feeding regimes to recapitulate the complexity of human obesity. We critically appraise the strengths and weaknesses of these different models to explore the underlying mechanisms, including the neural circuits that drive behaviours such as appetite control. We also discuss the use of these models for testing and screening anti-obesity drugs, beneficial bio-actives, and nutritional strategies, with the goal of ultimately translating these findings for the treatment of human obesity.

## Abstract (precis)

**Summary:** We review genetic models of obesity, their optogenetic and chemogenetic successors, and the use of dietary manipulations and meal-feeding regimes.

## Introduction

The scale of the global obesity epidemic demands a multidisciplinary response that utilizes all of the resources available to biomedical science. In 2014, the mean body mass index (BMI) of the world's population stood at 24 kg/m^2^, with approximately 39% of adults, aged 18 and over, being defined as overweight (with a BMI >25) and 13% as obese (BMI >30) [World Health Organization (WHO) Global Health Observatory; http://www.who.int/gho/ncd/risk_factors/overweight/en/). In addition, the prevalence of obesity has more than doubled since 1980 (http://www.who.int/mediacentre/factsheets/fs311/en/). Obesity is a major risk factor for three of the four main types of non-communicable disease (NCD; Box 1): cardiovascular disease (CVD; including heart attacks and stroke), diabetes, cancer, and chronic respiratory diseases [including chronic obstructive pulmonary disease (COPD) and asthma] (http://www.who.int/gho/ncd/mortality_morbidity/en/). NCDs contribute to more than half of all human mortalities, being the root cause of 38 million of the 56 million global deaths in 2012 (http://www.who.int/gho/ncd/en/). Because the overconsumption of energy lies at the heart of the increasing prevalence of obesity worldwide, improving our knowledge of the fundamental mechanisms of food intake and energy balance is important for developing effective strategies to influence dietary choice and to reduce energy intake to benefit the individual and to reduce the burden on healthcare systems and budgets.

**Box 1. Glossary**

**Anorexigenic:** reducing appetite or hunger, resulting in decreased food consumption and weight loss.

**Bed nucleus of the stria terminalis:** a fibre structure of the thalamus that links the amygdala with the hypothalamus and brainstem and has a role in anxiety and response to acute stress.

**Central nucleus of the amygdala:** a sub-region of the amygdala and its major output nucleus, projecting to and receiving input from sites involved in the stress response, including the hypothalamus, basal forebrain and brainstem, and with a role in the reception and processing of pain, fear and anxiety, and in mediating various behavioural and physiological responses to conditioned and unconditioned stimuli.

**Leptin:** a peptide hormone, primarily produced by white adipose tissue and in proportion to the size of adipose tissue stores, which induces satiety and reduces food intake.

**Mesolimbic reward system:** a dopaminergic brain pathway that connects the midbrain ventral tegmental area with the nucleus accumbens; dopamine released into the nucleus accumbens affects motivation for rewarding stimuli and perception of pleasure.

**Non-communicable disease (NCD):** a medical condition or disease that is non-infectious, i.e. cannot be transmitted from person to person; such diseases are often chronic in duration and progress slowly.

**Nucleus of the solitary tract:** also known as nucleus solitarius or nucleus tractus solitarii (NTS); brainstem site of integration of cardiovascular, respiratory, gastrointestinal and sensory information coming from peripheral organs, with onward transmission to the CNS to maintain homeostasis and influence neuroendocrine responses and behaviour, including feeding and reward.

**Orexigenic:** stimulating appetite or hunger, resulting in increased food consumption and weight gain.

**Parabrachial nucleus:** relay for taste, visceral sensation and hedonic information coming from the NTS for onward transmission to the hypothalamus and other forebrain regions.

**Periaqueductal gray:** grey matter located around the midbrain cerebral aqueduct with major functions including pain and analgesia, fear and anxiety, vocalization, and cardiovascular control.

Preclinical animal models are a key research tool in the quest to combat obesity, an effort that is likely to be ongoing for decades to come. One thrust of this research focuses on laboratory (mainly rodent) models of obesity. The currently available models are either genetic, such as spontaneous mutants or transgenic lines, or dietary, where the latter are the product of the same gene–environment interactions that underlie the majority of human obesity cases. Spontaneous-mutant and genetically engineered rodents provide valuable models of extreme obesity. Such models have been used with considerable success to examine the contribution of individual genes to adiposity and body weight regulation, and the role of different energy-balance signalling components (Zhang et al., 1994; Huszar et al., 1997). Despite the obvious importance of individual genes involved in energy homeostasis (e.g. Zhang et al., 1994), most cases of human obesity are polygenic rather than monogenic in nature, with contributions from different susceptibility genes. Progress has been made with the development of polygenic diet-induced obesity (DIO) models, but more work is required to better mimic human behaviours. This could include greater emphasis on dietary choices, meal-type feeding/drinking regimes, including binge-type eating, and investigation of motivation and compulsion to overeat. Obesity represents the outcome of gene–environment interaction, reflected in changed behaviour, yet all too often the environmental manipulation in preclinical models is relatively primitive, and might be restricted to a compulsory dietary change in isolation.

In this Review, we discuss various models of obesity, beginning with the traditional genetic models and the DIO models that involve intake of human foods, food choice, meal-feeding and binge-like eating. This outline includes consideration of key brain structures and neurochemical signalling components, with emphasis on energy-balance signals and neurodevelopment. With the advent of new molecular technologies such as CRE-*lox* recombination, CRISPR/Cas9 genome-editing, optogenetics and chemogenetics, mouse models have become more sophisticated and it has become possible to control the expression of specific genes both temporally and spatially (Huang and Zeng, 2013; Sternson et al., 2016). This has revolutionised the way we can interrogate the complex pathways involved in the control of energy balance. Thus, research has evolved from studies that primarily focused on understanding the basis of an obese phenotype to contemporary studies in which we can manipulate mouse genetics to decipher the neural circuits that control food-intake behaviour, the disruption of which ultimately leads to altered energy balance and obesity. These new models should advance understanding of relevant pathways. We discuss the strengths and weaknesses of the preclinical models and their potential to inform the evidence base and lead to the development of therapeutic interventions.

## Traditional genetic models of obesity

Human obesity is a complex genetic trait in which multiple genes and pathways contribute to overall energy balance. Genome-wide association studies (GWAS) have shown that the risk of obesity is determined by the cumulative contribution of many allelic variants (Speliotes et al., 2010), indicative of obesity being a polygenic trait. Despite this, the biggest advances in our mechanistic understanding of appetite and energy balance have stemmed from studies of monogenic mouse models. There are over 200 mouse models of monogenic obesity (Speakman et al., 2008; Lutz and Woods, 2012; Yazdi et al., 2015). Some of these arose as natural mutations (for example, the *ob/ob* mouse), whereas others have been genetically engineered, such as the melanocortin receptor 4 (*MC4R*) knockout mouse (Huszar et al., 1997), the tyrosine receptor kinase B (TrkB; also known as tropomyosin receptor kinase B) knock-in mouse (Byerly et al., 2013) and the FTO (fat mass and obesity-associated protein) overexpression mouse (Church et al., 2010). The most important obese model is the *ob/ob* mouse. This spontaneous mutant, discovered by The Jackson Laboratory (Mayer et al., 1951; Coleman, 1978), was key to the discovery of the leptin gene (Box 1), which is mutated in this mouse (Zhang et al., 1994). The discovery of leptin, and subsequently of the leptin receptors that are mutated in the *db/db* mouse (Tartaglia et al., 1995), focused attention on hypothalamic regions that are involved in integrating and controlling energy-balance signals (Schwartz et al., 2000). From this golden period of discovery came the description of complementary orexigenic and anorexigenic pathways (Box 1) (Schwartz et al., 2000).

**Fig. 1.**
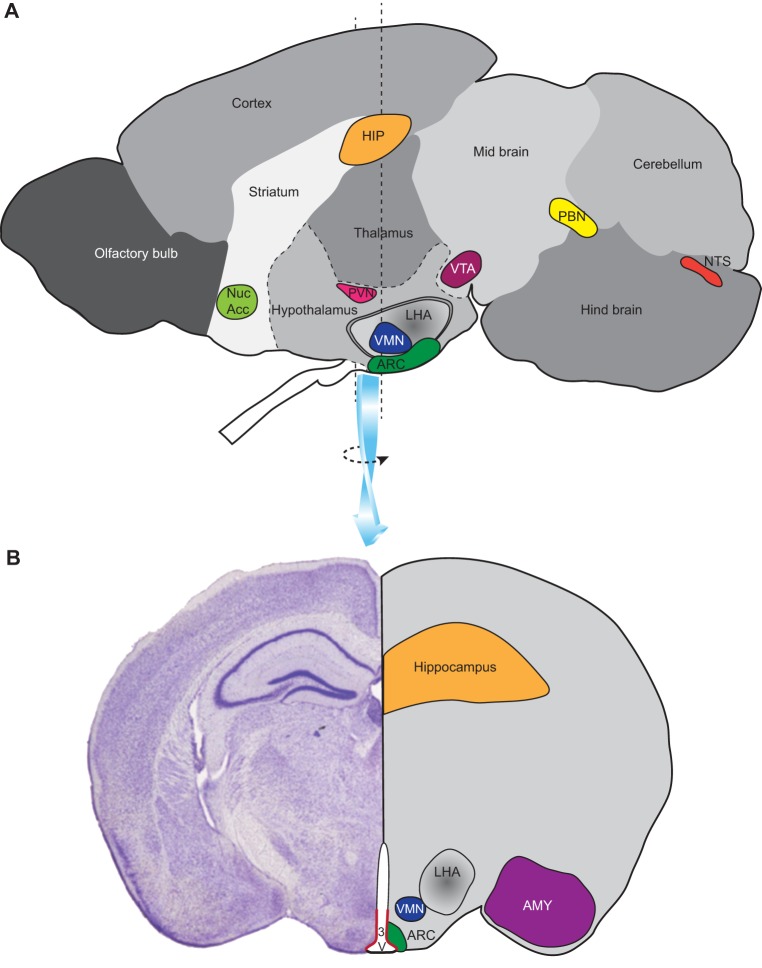
**Brain regions involved in food intake.** (A) A sagittal section through a mouse brain showing the relative positions of several regions (nuclei) of importance in aspects of food intake and energy balance: the nucleus of the solitary tract (NTS) and the parabrachial nucleus (PBN) are involved in receiving and relaying signals from the intestinal tract to other regions of the brain; the hypothalamic nuclei – the arcuate nucleus (ARC), ventromedial nucleus (VMN), lateral hypothalamus (LHA) and paraventricular nucleus (PVN) – contribute to the homeostasis of appetite and energy balance; the hippocampus (HIP) is involved in memory and learning associated with food intake; the ventral tegmental area (VTA; midbrain region) and nucleus accumbens (Nuc Acc; striatal region) are associated with registering the rewarding properties of food. (B) A coronal section through the brain in the region of the hypothalamus showing the relative positions of several aforementioned nuclei plus the amygdala (AMY), a region involved in memory and learning, and the ependymal layer surrounding the third ventricle (3V) where tanycytes located in the ventral third of this layer reside (highlighted in red).

The hypothalamus is a key region of the brain involved in nutrient sensing and homeostatic regulation. Important hypothalamic structures include the arcuate nucleus (ARC), paraventricular nucleus (PVN), ventromedial nucleus and lateral hypothalamic area (LHA), which have demonstrable roles in the homeostasis of appetite and energy balance (Rolls, 2005; Berthoud et al., 2011; Myers and Olson, 2012; Petrovich, 2013; Waterson and Horvath, 2015). The ARC (Fig. 1) harbours two distinct and important populations of neurons, one of which co-expresses agouti-related peptide (AgRP) and neuropeptide Y (NPY), leading to increased food intake, whereas the other expresses proopiomelanocortin (POMC) and cocaine- and amphetamine-regulated transcript (CART), resulting in reduced food intake. These neurons are key targets for leptin, which inhibits NPY/AgRP and stimulates POMC/CART neurons; the net effect of which is suppression of appetite (Cowley et al., 2001; Takahashi and Cone, 2004). Paradoxically, despite the strong orexigenic effects of NPY and AgRP when injected into the brain (Leibowitz, 1991; Marsh et al., 1999), *Npy-* or *Agrp*-null mice lack an overt food-intake or body-weight phenotype (Erickson et al., 1996). The double deletion of the *Npy* and *Agrp* genes also failed to induce a phenotype (Qian et al., 2002), whereas overexpression of *Agrp* led to obesity (Graham et al., 1997). The PVN is another important area, receiving neuronal projections from the ARC (including NPY/AgRP and POMC neurons) and elsewhere. From the PVN, projections run to the pituitary gland and extensively to other brain regions, including those with autonomic output to peripheral tissues (Ferguson et al., 2008). Via this pathway, NPY/AgRP and POMC/CART neurons contribute to the regulation of thermogenesis – a significant contributor to energy expenditure (Enriori et al., 2011; Shi et al., 2013). The melanocortin receptor 4 (*MC4R*) gene is strongly expressed in the PVN and, unlike in *Npy-* or *Agrp*-null mice, transgenic knockout of this gene results in an obese phenotype in mice (Huszar et al., 1997). In support of a role for this gene in human obesity, *MC4R* mutations are relatively common in obese individuals (Farooqi et al., 2003).

In addition to the hypothalamus, many other regions of the brain are involved in food intake (Fig. 1), including those involved in taste, olfaction, touch and vision, reward (such as the ventral tegmental area and nucleus accumbens) and learning and memory (hippocampus and amygdala), and hindbrain regions (the primary target of afferent signals from the gut following food ingestion), including the nucleus of the solitary tract (NTS; Box 1) and lateral parabrachial nucleus (PBN; Box 1). Tanycytes, a group of cells of emerging importance, are located at the interface between the third ventricle and hypothalamic neurons. These specialised cells form a component of the blood–brain barrier and might have a role in nutrient sensing and food intake, and function as progenitor cells for hypothalamic neurons (Bolborea and Dale, 2013; Pierce and Xu, 2010; McNay et al., 2012; Lee et al., 2012; Robins et al., 2013).

Although the concept of orexigenic and anorexigenic peptidergic pathways has provided a useful framework upon which to build our understanding of the central control of energy balance, more recent studies are starting to illustrate the limitations of this simple concept. Accordingly, focus in the field is now increasingly shifting from what we might consider as useful ‘mouse models of obesity’ towards the concept of ‘mouse models to understand obesity’, where the model may not itself be obese, but gives access to energy-balance mechanisms.

## Models of DIO

Changes in the human food environment (see Box 2) and the rise in the prevalence of global obesity have increased interest in rodent models of DIO and their applications. These models feature relatively subtle obesity phenotypes in which body fat accumulates over a relatively long time period, although the detrimental metabolic consequences of a high-fat (HF) diet are apparent within a few days (Williams et al., 2014). Compared to monogenic models, the timescales for development of DIO more closely mimic the gradual weight gain that occurs in much of the human population as a consequence of a marginally positive energy balance over many years; in this state, energy intake habitually exceeds energy expenditure, even if only by a small proportion.

**Box 2. The human food environment**

The global rise in prevalence of obesity over the last four decades in relatively (genetically) stable human populations emphasises that most human obesity represents the outcome of gene–environment interactions. Significantly, there has been a revolution in the human diet and in the wider environment during this period. Following on from major changes in agricultural production, towards mechanisation and intensification, there has been a near-complete transformation in food production and retailing, with extensive year-round product ranges largely replacing the traditional local and seasonal supply of food. Food processing has extended choice, improved overall quality and shelf-life, and facilitated lifestyle change. Evidence in support of this is provided by data indicating that 60-80% of food energy consumption in some European countries now derives from highly processed foods (Slimani et al., 2009). In the United States, the consumption of food prepared outside of the home, as a percentage of total energy intake, nearly doubled (to 32%) in the 30 years to 2008 (United States Department of Agriculture, 2012). These trends seem likely to continue. It is also important to note that this expansion of product ranges and global sourcing has been accompanied by a reduction in the real cost of food; the proportion of household disposable income spent on food in the United States fell from 17.5% to 9.7% between 1960 and 2007 (United States Department of Agriculture, 2014). This might in part reflect the low cost per unit energy of fats and sugars incorporated into many processed food products (Drewnowski and Darmon, 2005). The modern food environment, especially in the Western world, is characterised by meals and snacks, caloric beverages, foods with high palatability and high energy density, large portion sizes, comparatively low price, ready availability, and extensive choice and variety. All of these factors contribute to what are comparatively recent, yet major, changes in our diet – changes that influence gene–environment interactions.

**Fig. 2.**
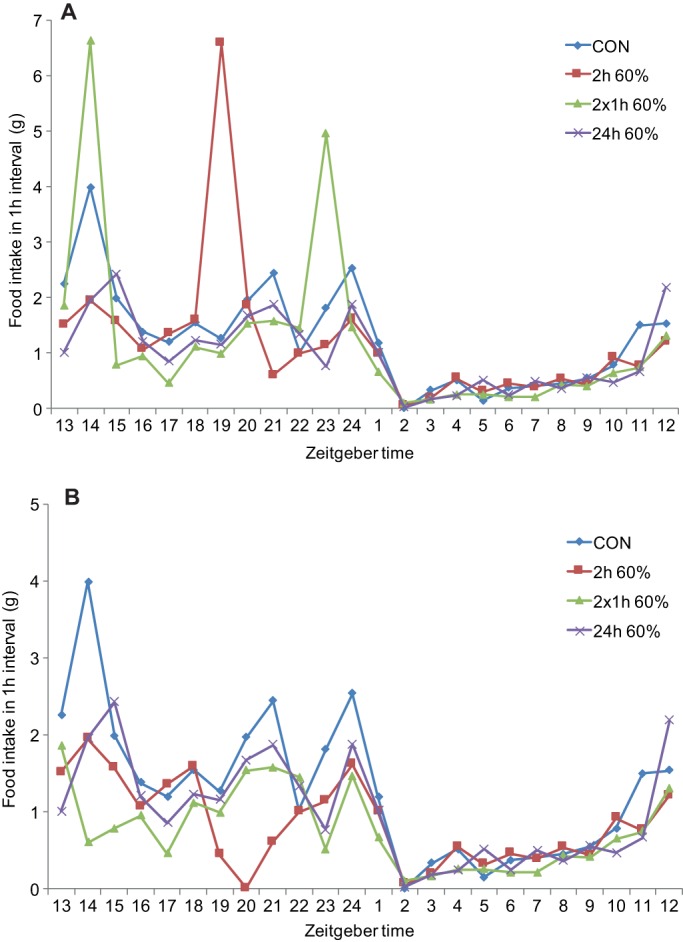
**Computerised food-intake monitoring from a binge-like eating study.** This figure illustrates findings based on unpublished data (T. Bake, D. G. A. Morgan and J.G.M.). Rats were given *ad libitum* access to: chow (CON); a 60% high-fat (HF) diet for 24 h per day (24 h 60%); or continuous access to chow and scheduled access to HF diet for 2 h in the dark phase, as either a single 2 h period (2 h 60%) or as two 1 h periods (2×1 h 60%). (A) Shows weight of food consumed, including from HF diet during scheduled feeding. (B) Shows food intake, excluding that from HF diet during scheduled feeding. These results show that: (1) intake is heavily nocturnal (ZT13-ZT24; zeitgeber time 0 is lights on in a 12:12 h light:dark phase that entrains biological rhythms) under all regimes; (2) rats given scheduled access to HF diet also consume chow during these periods; (3) rats consume a higher proportion of their daily caloric intake as HF diet when given 2×1 h access compared to a single 2 h scheduled feed; (4) compensatory reductions in chow intake were observed in the HF schedule-fed groups during the dark phase prior to and after HF binge-like eating.

At their simplest, rodent models of DIO involve switching animals from a relatively low energy-density diet that is low in fat and high in complex carbohydrates and fibre to one that is high in fat and sugar and is thus of higher energy density (Buettner et al., 2007). However, the simplicity of this model has drawbacks. A single, standardised, defined diet is still lacking, although many labs have gravitated towards defined diets, such as the 45% and 60% HF diets supplied by Research Diets, Inc. (D12451 and D12492). Examples of other diets used to induce obesity include a pure fat supplement to a chow diet (providing a measure of choice), or fat mixed with chow in a prescribed, no-choice proportion (example described in van de Giessen et al., 2012). A major drawback of DIO models is that data derived from their study can be inconsistent and irreproducible. The use of diets that vary in their macronutrient and micronutrient composition, and in energy density, consistency, flavour, physical formulation and palatability, gives rise to differences in energy intake, body composition and body weight trajectory *in vivo* (Buettner et al., 2007; Mercer and Archer, 2008). Furthermore, although many diets are relevant to (Western) human diets in composition, the animals are usually fed *ad libitum* throughout the light–dark cycle, often for periods of many weeks, and monotonously, without any choice. This default position is advantageous for the experimenter – there is no need to attend the research facility at regular intervals on a daily basis over extended periods to provide, withdraw or manipulate the diet, and experimental animals adopt a reasonably predictable pattern of food intake, concentrated in the hours of darkness (see Fig. 2). However, this only poorly replicates the meal-feeding choice habit of most human populations. Other dietary and feeding models that more closely mimic the human situation have been utilised preclinically and are discussed below.

### Cafeteria diets

So-called ‘cafeteria’ diets are superficially attractive as models of contemporary human obesogenic diets. These diets typically provide rodents with a mix of sweet and savoury, HF and/or high-sugar solid foods drawn from the human larder. Notably, in the pioneering studies of Rothwell, Stock and colleagues, rats were provided with four human food items per day, drawn from a selection of over 40 foods, creating a regime that resulted in increased energy intake and body fat content across a 15-day period in each of four different rat strains (Rothwell et al., 1982). However, such diets are used less widely than single-source HF diets, reflecting their logistical constraints, as well as concerns over the lack of dietary standardisation and variation in the diet selected, and the difficulty in calculating energy and macronutrient intake when diets are selected from a range of physically heterogeneous foodstuffs. Despite these potential issues, several investigators have recently revisited the cafeteria diet regime.

The ‘junk food’ diet, which provides a choice of processed foods and snacks in addition to a lard/chow mix, has been fed to adult Wistar rats of both sexes (Ong et al., 2013), to rat dams before being mated and until their offspring are weaned, and to their offspring as well (Ong and Muhlhausler, 2011). This diet and feeding regime resulted in increases in body fat mass in all groups tested. Perinatal exposure of offspring to HF, high-sugar diets increased their fat intake from the time of weaning, in a choice situation. This dietary manipulation also affected the expression of genes in the mesolimbic reward system (Box 1) (Ong and Muhlhausler, 2011). The expression of reward pathway genes (tyrosine hydroxylase, D1 dopamine receptor, µ-opioid receptor) was also affected in adult rats fed this diet (Ong et al., 2013). Chronically feeding adult male Sprague Dawley rats a chow diet mixed with lard and condensed milk, and supplemented with supermarket foods that are either high in protein and/or carbohydrates, or high in fat and sugar, also resulted in increased body weight (Martire et al., 2015). In addition, this cafeteria diet affected feeding behaviour (Martire et al., 2015) and faecal microbiota (Kaakoush et al., 2016). Reward mechanisms are likely to play a major role in cafeteria-diet-induced obesity. This was further evidenced by a study reporting that a cafeteria diet of sweet and savoury human foods led to weight gain and obesity in male Wistar rats (fed for 18-23 h per day over a 40-day period), and these metabolic effects were accompanied by compulsive-like feeding behaviour and downregulation of striatal D2 dopamine receptors (Johnson and Kenny, 2010).

### Fat or sugar choice diets

Cafeteria diets, given their high resource input and the heterogeneity of the foodstuffs used, are of limited use for routine high- or semi-high-throughput screening of potential therapeutics. Simplified versions of a cafeteria-type diet have therefore been developed, which offer choice but with less variety and a simpler formulation. The ‘free-choice high-fat high-sugar’ diet (la Fleur et al., 2014) allows rats to choose between a dish of saturated fat, a bottle of 30% sucrose, and standard chow. Animals were persistently hyperphagic (eating to excess) and became obese compared to those fed a no-choice pre-mix of fat, sugar and chow, which induced only transient overeating. Elevated caloric intake in the free-choice group was the result of an increase in the number of meals due to drinking of sugary liquid without a change in meal size. This obesity model has the attraction of inducing the overconsumption of calories while feeding to appetite on a choice diet. From a wider perspective, the ability to assess dietary preference and to quantify choice between dietary alternatives might be important for determining the consequences of complex interventions, such as early-life nutrition (discussed below). Many of the behavioural phenotypes that result from such interventions are likely to be quite subtle.

### Meal feeding

The generic model of *ad libitum*, completely free access to a single HF diet might be of value for screening pharmacological agents or other bioactives, but it does not easily relate to human diet and behaviour, any more than it does to that of rodents in their natural environment. An alternative to ‘free’ feeding to dietary excess from food available round the clock is to provide food for periods of time determined by the experimenter, i.e. to provide ‘set meals’. These access periods can be scheduled or randomised within the light–dark cycle and in the active or inactive periods of the animal, and thereby generate habitual or responsive meal-feeding behaviours. The marginal caloric overconsumption that lies at the root of much human obesity is based around choice and the provision of relatively regular meals, snacks and caloric beverages. Although meal-feeding animal models might thus better mimic patterns of human food intake, it still involves imposing a feeding pattern on the animal. In addition, the specific dietary manipulation might not lead to weight gain or to increased adiposity, depending on how long food is available for and how much energy is consumed. Nevertheless, imposed meal feeding has the advantage of standardising the physiological state of the animal prior to, during and after the meal because the amounts of energy ingested and the timing of meals are largely defined. Because excess weight gain might not be generated, meal feeding has been used to study hunger and satiety, and the brain response to food ingestion, rather than obesity. For example, this regime has been used to examine hypothalamic and brainstem activation in anticipation of, during and following 2 h of scheduled feeding on chow (Johnstone et al., 2006). In another study, rats were trained to receive a meal of set energy content for 1 h, and were later given *ad libitum* access to the same diet for 7 h (Chaumontet et al., 2015). This feeding regime ensured that animals ate a standardised meal (in terms of calorie content) during this 1 h period each day, in order to control their physiological state when meal tests were to be presented and their effects assessed. In another example, mice were allowed to consume two *ad libitum* meals of 45% HF diet (D12451), each of 2 h duration (Sasaki et al., 2015). The study applied variants of a feeding-plus-exercise regime and reported that a feeding time of 2 h prior to 2 h of exercise for each meal gave the greatest attenuation of HF-diet-induced weight gain.

### Binge-type feeding

The meal-feeding approaches outlined above are based on scheduled feeding where no nutrition is available at other times. Binge eating is defined in the Diagnostic and Statistical Manual of Mental Disorders (DSM-5; http://www.dsm5.org/) as the consumption of more food than is normal in a short time period, with a lack of control, and eating when not hungry. In preclinical animal models, it is effectively an extreme form of meal feeding. A review of different models of binge-like eating suggests that access to palatable food is necessary but not sufficient for bingeing behaviour to be expressed, that binge-like consumption of palatable foods requires intermittent (rather than continuous) access to the food, and that this non-homeostatic consumption can occur independently of obesity (Corwin et al., 2011). In a binge-like eating model in which continuous access to chow was supplemented for 2 h with vegetable fat, feeding behaviour was affected but not body weight or composition (Corwin et al., 1998). This model was further developed using the HF diet (D12451), again made available to rats for 2 h per day with access to a normal chow diet *ad libitum* for the remaining 22 h (Berner et al., 2008). This manipulation, in which greater than 50% of daily calories were consumed from the HF diet during the access period, increased 24 h caloric intake and body weight (Berner et al., 2008). We have extended this model to both 45% and 60% HF pellet diets (D12451 and D12492; Bake et al., 2013), to liquid palatable supplements (Ensure™ – a liquid nutritional supplement or 12.5% sucrose; Bake et al., 2013), and to mice as well as rats (Bake et al., 2013, 2014a). Rats that had access for 2 h to the 60% HF diet consumed 60% of their daily calories from this source, leading to elevated fat mass, whereas, under the same regime, the complete liquid diet, Ensure™, contributed 49% of their daily caloric intake (Bake et al., 2013). The capacity for binge-like eating on HF pellet diets was even more pronounced in mice (C57BL/6) than in rats. These mice consumed 86% of their daily calories during the 2 h scheduled access period, but in this case without increased body adiposity (Bake et al., 2013).

### Impact of maternal diet on offspring

In humans, maternal obesity contributes to a higher risk of obesity, metabolic dysfunction, type 2 diabetes and cardiovascular disease in offspring (Zambrano et al., 2016), and the proportion of women of child-bearing age or mothers-to-be who are overweight or obese is similar to the general population. Given the broad array of factors that regulate brain development, and particularly development of the hypothalamus [e.g. hormones, such as leptin; morphogens, such as Sonic hedgehog; and transcription factors, such as Sim1 (Biran et al., 2015; Blackshaw et al., 2010)], it is not surprising that nutritional status during development and early-life experiences, including those experienced *in utero*, affect developmental outcomes.

Although brain development follows a similar pattern in all mammals, it is not complete in rodents at the time of birth (Grove et al., 2005). Importantly, the hypothalamus continues to develop postnatally (Bouret and Simerly, 2006), facilitating studies of the roles of metabolic hormones, and the impact of maternal and foetal nutrition on the development of the neurocircuitry that regulates appetite and energy balance.

In this context, the identification of a stimulatory role for leptin in the development of appropriate neuronal projections from the ARC to the PVN was a key finding. These projections are disrupted in mice that lack functional leptin, and leptin treatment in adulthood was ineffective in re-establishing the innervation of the PVN from the ARC (Bouret et al., 2004). However, a rewiring of available connections could form the basis for the ability of exogenously administered leptin to reduce body weight (Pinto et al., 2004), and incomplete establishment of ARC–PVN connections might account for the progressive increase in fat mass during normal ageing (Breslow et al., 1999). Another hormone that functions in the development of the hypothalamus is ghrelin, a stomach-derived hormone thought to be involved in initiating meal feeding (Cummings et al., 2001; Steculorum et al., 2015). The presence of receptors for other metabolic hormones on hypothalamic progenitor cells indicates further potential influences on hypothalamic development and plasticity (Rodriguez et al., 2005).

In related studies, evidence indicates that a prenatal maternal HF diet has a detrimental effect on offspring, with increased numbers of neurons expressing orexigenic peptides such as galanin, dynorphin and enkephalin in the PVN and orexin and melanin-concentrating hormone (MCH) in the LHA. The increase in number of orexigenic neurons is not reversed when pups born to mothers fed HF diet are cross-fostered to mothers fed a chow diet, indicating a potentially permanent increase in appetite drive (Chang et al., 2008). Another mouse study has reported that a maternal HF diet during lactation detrimentally affected the projections (POMC and AgRP neurons) to key areas of the hypothalamus in offspring, including the PVN (Vogt et al., 2014). Total undernutrition also has consequences for neurogenesis and hypothalamic development, and can lead to a reduction in POMC and an increase in NPY neurons. These changes might result in hyperphagia and sensitivity to an obesogenic environment (Garcia et al., 2010; Ikenasio-thorpe et al., 2007; Desai et al., 2011). Although limited, studies investigating the impact of diet pre-conception, during gestation and during the post-natal period clearly demonstrate the importance of diet for foetal programming. Rodents will continue to be valuable models in which to determine the effect of maternal diet and obesity on physiological and molecular consequences in offspring. These models will be particularly useful in unravelling the role of epigenetics on the appetite and energy-balance regulatory system (Box 3).

**Box 3. Nutrition and epigenetics**

Epigenetic regulation of gene expression encompasses histone methylation and acetylation, which affect the compactness of chromatin and DNA methylation, specifically at CpG dinucleotides within regulatory DNA sequences. These are reversible physical modifications affecting the ability of transcription factors to bind to regulatory sequences that affect gene transcription. DNA methylation is likely to be more relevant to the developmental impacts of diet because CpG methylation patterns are highly stable and transmitted post-mitotically (Cedar and Bergman, 2009). A growing body of evidence suggests that diet might affect the epigenetic regulation of gene expression (Waterland, 2014). For example, maternal high-fat (HF) diet, which has an impact on a number of behavioural and physiological outcomes in offspring, induces differential DNA methylation in reward regions of the brain, with sex differences being observed (Carlin et al., 2013). The importance of further research on the susceptibility of developmental processes to dietary manipulation is highlighted by the potential impact of supplementing conjugated linoleic acid (CLA), a nutrient with suggested health benefits (Benjamin and Spener, 2009), during pregnancy. In a rat model, supplementing the maternal diet with CLA during the weaning period promoted the hypermethylation of the *POMC* promoter and decreased POMC expression in the hypothalamus of suckling offspring, which contributed to retarded growth during weaning and to a larger body mass with associated metabolic disorders after weaning (Zhang et al., 2014).

### Behavioural phenotyping using models of DIO

As outlined above, evidence is accumulating that nutritional experience early in life (*in utero* and neonatal) and epigenetic gene regulation (Box 3) can influence later diet choice (Brenseke et al., 2015). It is therefore important to accurately assess behavioural phenotypes in obesity models. However, many preclinical studies still limit food-intake data to extended periods (hours or days), providing little information about meal patterns and temporal characteristics. Computerised systems such as the CLAMS (Comprehensive Lab Animal Monitoring System; Columbus Instruments) or PhenoMaster/LabMaster (TSE Systems) systems can measure the weight of solid or liquid food consumed on a minute-by-minute basis using either a suspended feeder or an under-floor balance. Feeding data can be assembled in suitably sized data bins to allow feeding to be measured with precision and in a timeframe relevant to other behaviours, such as physical activity. The utility of computerised systems is illustrated in the study depicted in Fig. 2 (Bake et al., 2014b; TSE Systems), where rats were provided with chow diet *ad libitum* 24 h per day, and a 60% HF diet (D12942) on a scheduled feeding regime for 2 h per day in the dark phase as either a single 2 h period or two 1 h periods. Another group was fed a HF diet *ad libitum*. Access to the palatable diet was automated using a retractable sleeve mechanism thereby eliminating operator disturbance. With simultaneous monitoring of intake of each diet across a 24 h period, and with data presented as 1 h bins at a modest level of granularity, considerable detail can be observed; for example, which dietary components are selected and when (when a choice is available), and over what timescale are compensatory adjustments made for the excessive caloric intake during limited access to the palatable diet (Fig. 2).

The dietary manipulations and feeding regimes outlined above are frequently used to induce obesity or feeding behaviours of interest in otherwise normal rodents. These animals can be drawn from inbred lines with similar genetic backgrounds or from outbred lines that have the potential to present a range of susceptibilities to DIO. In such outbred lines, exposure to an obesogenic diet can give rise to a range of body-weight trajectories reflecting relative susceptibility or resistance to DIO, a situation assumed to reflect human polygenic obesity. The various diets and feeding regimes can also be used as tools to manipulate spontaneous genetic and genetically manipulated models of obesity to interrogate specific mechanisms involved in the gene–environment interplay underlying obesity.

## Modulation of genetic models: new approaches

As described earlier, transgenic knockout animals have been useful in defining some of the genes involved in the neural control of energy balance (e.g. *MC4R*). Yet, even with targeted deletions, such models are unable to provide a complete understanding of the specificity and connections involving these genes. The recent development of optogenetic and chemogenetic mouse models in combination with imaging techniques is opening up the possibility to understand and decipher the complex wiring of the brain and relate this to behaviour (Huang and Zeng, 2013; Sternson et al., 2016).

### Optogenetics and chemogenetics: an overview

The major advance that optogenetics affords is the ability to create mouse models in which a small group of neurons can be activated or inhibited using light (Nagel et al., 2003; Boyden et al., 2005; Atasoy et al., 2008). This technique relies on the ability to express light-activated ion channels, such as the algal opsin channelrhodopsin-2 (ChR2), in a restricted set of neurons and then to switch these neurons on or off using light delivered locally to the region of expression (Atasoy et al., 2008; Ellenbroek and Youn, 2016). A key technical issue is the ability to express the ion channel in the target neurons with the sufficient level of expression and specificity. ChR2, for example, has only single-channel conductance and therefore needs to be highly expressed to be effective. To achieve such targeted and high-level expression, an intersectional approach is used (Huang and Zeng, 2013; Sternson et al., 2016). Specificity is provided using the Cre recombinase system. Mouse models are engineered to express Cre recombinase under the control of cell-type-specific promoters, and stereotaxic injection of a Cre-dependent virus [e.g. adeno-associated virus (AAV)] with a strong promoter drives the high-level expression of the light-sensitive ion channel. Using this approach, only those cells expressing Cre recombinase also express the light-sensitive ion channel. To activate these cells, light of a specified wavelength is delivered to the target area in the brain through an optic fibre that is stereotaxically positioned through a guide cannula in close proximity to the target cells. Although the use of an optic fibre, which must be connected to a light source, limits the totally free movement of the animal, it nonetheless allows for very precise control of a small subset of neurons.

The ability to either stimulate or inhibit neuronal activity can be determined by the choice of light-activated ion channel (Huang and Zeng, 2013). Cation-conducting opsins, which include ChR2 and the channelrhodopsin variants ChIEF and CHETA, cause depolarising activation of neurons and are activated by blue light. Although red-light-activated opsins (VChR1 from the colonial algae *Volvox carteri*, and the ChR chimera, C1V1) are also available, ChR2 has been the most commonly used stimulatory light-activated ion channel to date, probably because it was the first light-activated channel to be used to excite neurons (Boyden et al., 2005). Light-activated anion (e.g. chloride) channels are used to hyperpolarise (inhibit) neurons. The most commonly used among these is halorhodopsin, a chloride pump that is activated by yellow light. A key benefit of optogenetics is that it provides targeted and precise cell activation, over millisecond timescales, in non-sated, freely moving animals. This allows questions to be asked about the functional relationship between specific neurons or neuronal subsets and feeding behaviour, transforming how we can use animal models in obesity research.

Chemogenetics involves the targeted introduction of an engineered gene into specific cell types in mice to achieve high-level and targeted expression. The engineered gene can be either a modified G-protein-coupled receptor (GPCR) or an ion channel. Then a drug, rather than light, is used to activate or to inhibit neurons of interest. The approach used most often to date in studies of appetite circuits involves the use of a DREADD (designer receptor exclusively activated by designer drug) (Huang and Zeng, 2013; Sternson et al., 2016). These are based on GPCRs that have been engineered to respond to ligands that are considered inert in mammalian systems. For activation, the DREADD hM3Dq is used, which is a modified Gq-coupled muscarinic receptor that is activated by the largely inert compound CNO (clozapine-N-oxide) (Conklin et al., 2008). For inhibition, hM4Di is used, which is a modified Gi-coupled muscarinic receptor, also activated by CNO (Armbruster et al., 2007).

### Using optogenetic and chemogenetic models to study appetite

The use of these innovative techniques brings a new temporal and spatial dimension to understanding how feeding circuits in the hypothalamus function. Recent chemogenetic studies have resolved some of the paradoxical outcomes of conventional gene knockouts (e.g. *Npy*, *Agrp*) discussed earlier. To selectively destroy AgRP/NPY-expressing neurons (Luquet et al., 2005), mice were engineered to express the diphtheria toxin (DT) receptor under the control of the *Agrp* promoter. Ablating these neurons with DT during adulthood caused anorexia, whereas there was minimal effect on feeding if ablation was induced during the neonatal period (Luquet et al., 2005). In line with the ability of leptin to rescue development of hypothalamic projections in leptin-deficient neonates (Bouret et al., 2004), compensatory mechanisms are thus able to be recruited following the ablation of AgRP/NPY-expressing neurons in neonates but not in adults, further emphasising the importance of early-life stages and their potential for manipulation to negative or positive effect.

More recently, a combination of optogenetic and chemogenetic studies has shed new light on the functionality of the AgRP/NPY neurons. Both optogenetic and chemogenetic activation of ARC AgRP neurons leads to strong food-seeking behaviour and to food consumption within a few minutes of activation (Aponte et al., 2011; Krashes et al., 2011). This could be blocked by injection of an antagonist of either NPY receptor 1 or GABA_A_ (γ-aminobutyric acid) receptor into the PVN, indicating that both NPY and GABA, released by the AgRP/NPY neurons, are required for acute feeding responses (Atasoy et al., 2012). By contrast, feeding responses were maintained when AgRP/NPY neurons were chemogenetically activated in either *Npy*-null mice or in mice selectively lacking *GABA* in AgRP neurons (Krashes et al., 2013). A likely explanation lies in changes in signalling in the *Npy*-knockout mice. In line with this, synaptic output associated with GABA release was found to be increased (Atasoy et al., 2012). Taken together, these data show that both NPY and GABA are required for the acute feeding response involving AgRP/NPY neurons in the ARC. They also suggest that compensatory mechanisms are likely to contribute to the observed lack of effect in knockout animals.

One of the benefits of using photostimulation in optogenetics is the ability to define the temporal nature of a response. Sternson and colleagues used this approach to study the temporal dissociation of feeding responses involving AgRP, NPY and GABA neurons. Whereas NPY and GABA are involved in acute feeding responses over minutes, the feeding response to AgRP is delayed for hours, revealing that AgRP has a discrete function relative to either NPY or GABA. Thus, optogenetic and chemogenetic studies can reveal the functional complexity within a single neuronal cell type. Perhaps more fundamentally, they also highlight how findings based on animal models derived from mutation or knockout of a single gene can be misleading.

### Activating feeding circuits with electromagnetic waves

An interesting new development is the use of electromagnetic radiation (radiowaves or magnetic fields) to either activate or inhibit ion channels to control feeding and glucose-sensing in the mouse hypothalamus (Stanley et al., 2016). Again, a key requirement is to have mouse lines that express Cre recombinase in targeted neurons. These can then be injected with a replication-deficient adenovirus that expresses ferritin nanoparticles tethered to a temperature-sensitive cation channel, TRPV1 (transient receptor potential vanilloid 1), which allows targeted neurons to be activated via the controlled entry of Ca^2+^. Inhibition of neuronal activity can be achieved using a mutated form of TRPV1 that is anion (chloride) permeable. An advantage that electromagnetic waves have over an optogenetic approach is that no permanent implants (e.g. optic fibres) are required to deliver the stimulus to activate or inhibit the neurons of interest, and the approach lends itself to the activation of more dispersed neuronal groups than can be achieved when using a light source. Yet, like optogenetics, it also allows neurons to be more rapidly activated and inhibited than is possible using chemogenetics (Stanley et al., 2016).

### Exploring neural connectivity using optogenetics and chemogenetics

Optogenetic and chemogenetic approaches have also been harnessed to define new and sometimes unexpected pathways and connectivity relevant to appetite control. For example, cholecystokinin (CCK) neurons expressed in the NTS of the hindbrain have been shown to suppress hunger even in fasted mice, through independent projections to the PVN and the PBN (D'Agostino et al., 2016; Roman et al., 2016). Chemogenetic and patch-clamp studies showed that the stimulation of NTS CCK neurons increases fos immunoreactivity and that neurons project to MC4R-expressing cells in the PVN (D'Agostino et al., 2016). Stereotaxic injection of a Cre-inducible AAV vector that expresses ChR2-eYFP into the NTS of CCK-iCRE recombinase mice, combined with stimulation of the PVN with blue light, then revealed a functional connection between the NTS and PVN through CCK neurons, which suppresses food intake (D'Agostino et al., 2016). Similar approaches have also shown that CCK and noradrenergic, dopamine β-hydroxylase (DBH)-expressing NTS neurons provide two separate projections that directly excite calcitonin gene-related protein (CGRP)-expressing neurons in the PBN and that these also mediate anorectic effects (Roman et al., 2016).

Optogenetic approaches have also been used to define the projections and responses of AgRP neurons within the ARC (Atasoy et al., 2012; Betley et al., 2013, 2015). These studies showed that optogenetic activation of AgRP projections to the PVN, bed nucleus of the stria terminalis (Box 1) and LHA stimulate increased food intake, whereas projections to the central nucleus of the amygdala (Box 1), periaqueductal gray (Box 1) and PBN did not. These results together with those in the NTS emphasise how a number of distinct neural circuits contribute to the feeding response and that this behaviour might involve only a small subset of a specific neuronal type, underscoring the specificity of neuronal projections involved in a given physiological response.

Another important facet of these new approaches is that they have revealed how sensory state can rapidly influence the activity of these feeding circuits. For example, optogenetics has been used for deep-brain calcium imaging to provide a read-out of neural activity of hypothalamic AgRP and POMC neurons (Chen et al., 2015; Betley et al., 2015). These studies have shown that sensory detection of food, even before consumption, can rapidly reset the activation state of AgRP and POMC neurons (Chen et al., 2015; Betley et al., 2015). Thus, these new technologies are providing new animal models that take us way beyond the single-gene-mutation models of obesity, and are starting to give us a glimpse into real-time control of food-intake behaviour through temporally and spatially defined neural circuits.

## Strengths and limitations of preclinical obesity models

In assessing the comparative strengths and weaknesses of the models described above, a key consideration is what we are trying to achieve in generating and studying these models. Is the objective to generate an obese phenotype in a suitable rodent model that can then be used to characterise differences with non-obese equivalents, or to screen dietary or drug interventions? Or is it to generate more sophisticated *in vivo* tools that could be used to resolve some of the fine details of energy balance and appetite mechanisms? If the former, how relevant are these rather simple models to human obesity, a multifaceted condition with infinite root causes, trajectories and outcomes? Another limitation could be the tendency of experimenters to move obese animals towards body-weight and body-composition extremes in order to maximise the contrast with normal-weight controls, when more subtle phenotypes are likely to be more realistic. If the objective is to generate *in vivo* tools, is it important to note that many such models are not actually obese, and the molecular manipulations employed might not induce obesity.

Although recent reports, outlined above, have begun to move dietary obesity models towards manipulations that more closely mimic the human situation (developments that should be encouraged), the manipulations and the measures being made remain mostly rudimentary. For example, a stark contrast between a very bland diet, very low in both fat and sugar, and a palatable/hyperpalatable diet, both fed *ad libitum*, is not one that most humans will experience. Everyday experience in developed countries can probably be summed up as a fundamentally palatable varied diet superimposed with hyperpalatable dietary components that are likely to be familiar to the consumer and elicit learned responses. Such a regime would be valuable to replicate in a preclinical model, with the goal of inducing mild DIO and periodic hyperphagic or binge-like eating episodes. As well as the diet itself, it is also difficult to effectively model context, such as setting and varying levels of hunger, key components of the human food experience. Even when relatively complex, varied diets are employed, often only food-intake data are collected, and usually only across limited sampling periods of a few hours. Furthermore, the other side of the energy-balance equation, expenditure, is only rarely monitored as either total energy expenditure (by calorimetry) or as physical or locomotor activity. A sophisticated model of obesity might also build in graded levels of sedentariness or activity, but again there would be a need to move beyond a binary choice of extremes of activity level.

The strength of monogenic models of obesity has been in the identification of specific genes and their role in the regulation of energy balance. At their best this has given us a basic insight into regulatory pathways of food-intake behaviour. A good example of this is leptin signalling through the orexigenic (AgRP) and anorexigenic (POMC) neurons in the ARC and the MC4R-expressing cells of the PVN. Despite success in identifying hundreds of genes that are important to energy balance, no single molecular target has led to the creation of a blockbuster drug for obesity. This is because discrete neural circuits rather than single molecular targets underlie the control of food-intake behaviour. Furthermore, where specific molecular targets are important to food-intake behaviour and obesity, they are also important to other behaviours and physiological responses, and targeting these gives rise to undesired side effects. For example, MC4R is clearly important to target inhibition of food intake, yet activation of this receptor is also thought to stimulate penile erection (Adan et al., 2006). This underscores the need for new models of obesity that enable both temporal and spatial dissection of the neural circuits involved in food-intake behaviour and obesity. From the more sophisticated tools that we now have, a more comprehensive picture of the neural pathways involved is emerging, and this is taking us beyond the simple ‘one-drug-targeting’ and ‘one-receptor-subtype’ concepts to address obesity as a behavioural deficiency. In turn, this is challenging us to think more radically about drug development in the future, as discussed below.

## Therapeutics and translation: the future for preclinical obesity research

Preclinical research into obesity has the potential to drive the development and characterisation of dietary interventions, as well as the identification of drug targets and viable pharmacological agents. Importantly, adoption of either dietary or pharmacological approaches in a clinical setting is usually accompanied by the recommendation of lifestyle change, an aspect that is not easily mimicked in experimental animals.

Diet-based strategies for obesity and weight management include weight loss in the obese, where even small amounts of weight loss can be very beneficial for long-term health, and maintenance of lower body weight following weight loss achieved by caloric restriction. For overweight individuals who might be on an upward trajectory towards obesity if they cannot limit future weight gain, prevention of weight gain is a viable target. Suitable dietary manipulations for such individuals, such as high-protein or high-fibre diets and the hunger and satiety mechanisms that regulate weight gain, can be studied in preclinical models. The mechanisms involved in hunger and satiety and that could be targeted by diet might encompass gut hormones, short-chain fatty acids and the gut microbiota, and onward signalling from the periphery to the brain. It is not yet clear to what extent the effectiveness and sustainability of dietary interventions and their outcomes in a clinical context will depend upon an individual's position within the BMI range, i.e. what diets fit which phenotype (or genotype). The likelihood that ‘one size will not fit all’ could certainly be examined in suitable preclinical models.

The preclinical models generally employed directly in drug testing can be broadly categorised as genetic or DIO (Cheetham and Jackson, 2012; Nilsson et al., 2012), or monogenic or polygenic, as described above. Again as outlined earlier, the focus of drug effects is mainly food intake, the most common pharmacological target for anti-obesity drugs, although secondary metabolic disease is also of therapeutic interest. Candidate drugs are generally tested in mice and rats, and the effects of novel agents are usually compared to those of known therapeutics. This might form a starting point for integrating more subtle models and dietary manipulations into a testing regime.

Development of therapeutics for the treatment of obesity has relied upon traditional approaches to drug development. This has involved targeting specific receptors or enzymes, where the specificity of action relies mainly on the molecular subtype(s) involved in the physiological response. Invariably, this offers a low level of discrimination; thus, anti-obesity therapeutics often elicit many unwanted side effects. Also, the behaviours involved, such as appetite control, are complex and involve neuronal circuits rather than single molecular targets, meaning that drugs rarely achieve the level of therapeutic efficacy and benefit desired. For these reasons, many drugs aimed at the obesity market have ultimately been withdrawn from the market after only a short period (Kang and Park, 2012). Animal models that involve the use of technologies such as optogenetics and chemogenetics to explore the neural circuits that control behaviours relevant to obesity are leading to new ways of thinking about the development of therapeutics, as well as new methods of delivery. Traditionally, drug development has approached drug design by targeting what nature has to offer (e.g. receptors). In the future it might be possible to design the target as well as the drug, where the aim will be to introduce the target into specific neural circuits using gene therapy approaches, and then manipulate this construct. There are some important and big challenges to overcome to make this a practical reality for the patient, but the new animal models involving opto- and chemo-genetics to explore the neural circuits underlying obesity are key to defining where and how the designer targets are located.

The future for preclinical models in obesity research should also see more sophisticated phenotyping of feeding behaviours, including food choice and intake microstructure, being applied across a range of monogenic and polygenic obesity models, as well as to contemporary genetically manipulated animals designed to give access to detailed molecular and neural energy-balance mechanisms. These approaches will be particularly important for successful characterisation of the outcomes of subtle changes, including those occurring at an epigenetic level as a consequence of nutritional programming events (see Box 3). A concerted effort should be made to better mimic the human food experience (see Box 2).

All too often, research reports conclude that differences in experimental findings between similar studies from different laboratories are likely to reflect differences in experimental detail – e.g. the precise diet or feeding manipulation. This seems a poor outcome after decades of research. Food choice and meal-feeding scenarios should be carefully considered in all experimental designs, and there would be wisdom in the scientific community agreeing on a range of standard diets and manipulations in order to reduce inherent variability. Given a suitable level of standardisation of experimental variables, a database of environmental, behavioural and dietary characteristics and of related experimental outcomes would permit profitable meta-analysis, thereby maximising the value of related studies. The relative naivety of current approaches to studying the influence of diet, choice and dietary components on obesity and energy homeostasis are unhelpful for advancing our mechanistic understanding of these complex processes and for developing therapeutics. Over the last decade, the field of obesity research has moved on from an over-reliance on knockout rodent models with an obese phenotype towards more refined and subtle contemporary models that help us to elucidate the complexity and redundancy built into the neural circuits involved in the control of food intake and energy balance. The future of preclinical research in obesity should be based on more realistic models of feeding and obesity, better characterisation of feeding behaviour, and the application of molecular and genetic tools to advance our understanding of these complex mechanisms, how they are interlinked, and how they can be exploited for therapeutic benefit.

## Notes

This article is part of a special subject collection ‘Spotlight on Rat: Translational Impact’, guest edited by Tim Aitman and Aron Geurts. See related articles in this collection at http://dmm.biologists.org/collection/rat-disease-model.
